# The Role of microRNA in the Prognosis and Diagnosis of Ovarian Cancer

**DOI:** 10.3390/ijms26073413

**Published:** 2025-04-05

**Authors:** Mateusz Kozłowski, Dominika Borzyszkowska, Anna Golara, Jerzy Lubikowski, Aneta Cymbaluk-Płoska

**Affiliations:** Department of Reconstructive Surgery and Gynecological Oncology, Pomeranian Medical University in Szczecin, Al. Powstańców Wielkopolskich 72, 70-111 Szczecin, Poland

**Keywords:** ovarian cancer, cancer, prognosis, diagnosis, biomarker, marker, survival, microRNA, miRNA

## Abstract

Ovarian cancer (OC) is one of the most common cancers in women. Biomarkers for OC are still being sought. The aim of this review was to evaluate microRNAs in the prognosis and diagnosis of OC. We conducted a literature review searching for articles published from January 2014 to September 2024. We included articles presenting the association of microRNAs with ovarian cancer prognosis, where patient survival was shown by the Kaplan–Meier curve, and articles presenting the association of microRNAs with ovarian cancer diagnosis, where the results were presented as an ROC curve. MicroRNAs are promising clinical markers in ovarian cancer patients. As is shown here, expression (high or low) of various miRNAs was differentially associated with survival in OC patients, with some miRNAs being associated with a longer survival and some with a shorter survival. In the absence of diagnostic markers for OC, the raised role of miRNAs in diagnosis seems all the more important. The diagnostic value of miRNAs has been shown, mostly as blood biomarkers, although they have also been evaluated as tissue or urine markers. MiRNAs have an important role as clinical biomarkers for ovarian cancer, not only as single molecules, but also as biomarker pairs or panels of miRNAs. It should be noted that most of the miRNAs reviewed here have been studied once, so despite the promising results, it seems necessary to conduct studies to confirm or negate the results obtained.

## 1. Introduction

Ovarian cancer (OC) is the eighth most common and fifth most lethal cancer among women worldwide. Despite being the third most common gynecological cancer after uterine and cervical cancers, this illness is more deadly [1,2]. OC has three main histopathological types (epithelial ovarian cancer, germ cell tumors, and sex cord and stromal tumors), which differ in pathogenesis, biological behavior, histological morphology, clinical presentation, treatment methods, and prognosis. According to statistics, approximately 93% of patients can live longer than 5 years after diagnosis if the disease is detected with a small tumor volume or in a localized stage (stages IA and IB) [3,4]. Therefore, the development of a sensitive and specific biomarker enabling early diagnosis and screening for OC is of great importance. Diagnostics include a physical examination and transvaginal ultrasound (TVUS). Unfortunately, the screening strategy for the earlier detection of OC still requires further research and standardization. The most commonly used biomarker in clinical practice for detecting ovarian cancer is cancer antigen 125 (CA125), but it has low specificity. Multi-marker panels are also used, combining molecular biomarkers such as human epididymal secretory protein 4 (HE4), ultrasound findings or menopausal status, the ovarian risk of malignancy algorithm (ROMA), the risk of malignancy index (RMI), and tests. Research is also being conducted on new biomarkers such as autoantibodies, ctDNA, miRNA, and DNA methylation signatures that could enable the early detection of ovarian cancer. The following are considered to be prognostic factors in ovarian cancer: the stage of ovarian cancer, the histological type of the tumor, the degree of histopathological differentiation, the size of lesions left after cytoreductive surgery, the patient’s age, and the *BRCA1* or *BRCA2* mutation carrier status. Patients with OC are treated with a variety of techniques, including surgery, radiation, targeted therapy, hormone therapy, immunological therapy, and polyadenosine diphosphate ribose polymerase inhibitor maintenance therapy [5]. First-line therapy for ovarian cancer involves a combination of cytoreductive surgery and platinum-based chemotherapy [5].

MicroRNAs are RNAs that are approximately 18–24 nucleotides in length. They belong to the class of small non-coding RNAs and play an important role in the post-transcriptional regulation of gene expression, cellular metabolic pathways, and developmental events [6]. MiRNA biogenesis consists of multiple cleavage steps in the nucleus and cytoplasm. In the nucleus, microprocessor, a catalytic complex consisting of Drosha and Di George critical region 8 (DGCR8), cleaves the primary (pri)-miRNA transcript [7]. Through Drosha’s interaction with the basal UG motif and the alignment of the DGCR8 dimer with the apical UGU motif, stem-looped pri-miRNA is appropriately orientated for cleavage [8]. Then, the precursor (pre)-miRNA formed by microprocessor cleavage is transported to the cytoplasm by exportin-5 [9], where DICER1 cleaves the pre-miRNA [10]. The double-stranded mature miRNA is then bound by Argonaute (AGO) [11]. While the passenger strand, known as miRNA*, is cut off and destroyed, the guide strand stays linked to AGO to create the miRNA-induced silencing complex (miRISC) [12]. The RNA interference (RNAi) pathway is made possible by miRISC. As a result, the complementary Watson–Crick binding sites in the 3′UTR of the mRNA are recognized by the miRNA seed region, which spans nucleotides 2–8 from the 5′ end [13,14]. Post-transcriptional gene regulation is carried out by miRNAs. They interact with circular RNAs (circRNAs), long non-coding RNAs (lncRNAs), and pseudogenes to either increase cellular competition for miRNA binding sites or induce miRNA suppression [15]. MiRNAs can be detected in body fluids, as well as extracellular vesicles (EVs) and the tissue microenvironment. While larger vesicles, such as microvesicles or oncosomes, carry larger RNAs along with a larger proportion of miRNAs, exosomes only contain a small percentage of circulating miRNAs [16]. Changes in miRNA expression may influence the extent of target regulation and thus affect cell homeostasis [17]. Therefore, changes in miRNA, and consequently mRNA, play an important role in carcinogenesis (from the initiation stage to the formation of metastases) and other diseases. Deregulation of the level of various miRNAs has been observed, for example, in colorectal cancer, breast cancer, lung cancer, and ovarian cancer. Selected miRNAs can be found and their expression profiles can be tracked to aid in the early diagnosis of cancer cells and to predict how the disease or therapy will progress.

The aim of this review was to evaluate microRNAs in the prognosis and diagnosis of ovarian cancer. We conducted a literature search on PubMed. We searched for articles by the phrases ’ovarian cancer’ and ’microRNA’. We wanted to focus on scientific reports of the last ten years, and therefore searched for articles published from January 2014 to September 2024. For the review, we included articles in English, with access to the full text, presenting significant results for specific microRNAs. We included the following: (1) articles presenting the association of microRNAs with the prognosis of ovarian cancer, where the survival of patients was presented with a Kaplan–Meier curve, and (2) articles presenting the association of microRNAs with the diagnosis of ovarian cancer, where the results were presented as an ROC curve. Review articles were excluded from the review. Eligible articles are presented in Section 2 and Section 3 of this manuscript.

## 2. The Role of miRNAs in the Prognosis of OC

As described, miRNAs are involved in numerous cellular processes. A particularly well-known role is the regulation of gene expression. Undoubtedly, there is also a need for research on these molecules, taking into account the clinical aspect in order to assess the prognosis of patients with ovarian cancer. Here, we review the literature assessing how the expression of particular miRNAs is related to the survival of ovarian cancer patients. MiRNAs are a large group of molecules that are being researched all the time, especially in cancer. Therefore, most miRNAs are described once in the literature. The most clinical information is provided by miRNAs, which have been described many times. To date, the miRNA-200 family seems to be the best described. Comparing the high vs. low expression of miRNA-200b, it was clearly shown that high expression was associated with a shorter overall survival [18,19,20]. A study by Zhang et al. also showed a shorter disease-free survival in patients with high miRNA-200b expression [18]. Higher miRNA-200a expression was also associated with a shorter OS (overall survival) and DFS (disease-free survival) [18]. In contrast, the results of miRNA-200c studies are inconclusive. On the one hand, a shorter OS and DFS were found in patients with a high expression [20], while another study showed better OS [21]. Two studies showed that high miRNA-145 expression was associated with better OS [22,23]. Unambiguous results indicating that higher expression was associated with better survival were also shown for miRNA-23b [24,25]. For miRNA-25, on the other hand, the data are inconclusive. Li et al. showed better OS and PFS (progression free survival) in patients with higher expression [26], while a study by Wang et al. found a shorter OS with higher miRNA-25 expression [27]. As we mentioned earlier, most of the miRNAs have been described once, so we have listed their prognostic value collectively in Table 1. Since epigenetics and especially miRNAs are such an intensely developing science, it is important to look for changes specific to particular subtypes/subgroups of ovarian cancer. Most of the studies reviewed here focused on the ovarian cancer/epithelial ovarian cancer group in general. Nevertheless, Biegała et al. described a group of serous ovarian cancer patients in which, at higher expression, they found better survival for miR-99b-5p, miR-505-5p, miR-424-3p, miR-324-5p, and a shorter survival for miR-100-5p and miRNA-125a-3p [28]. A more defined group was also described by Wilczyński et al., where they found better survival in advanced serous ovarian cancer patients with higher miR-146a expression [29]. Similarly, Kim et al. studied high-grade ovarian serous carcinoma patients, where they found better OS in patients with higher miR-145 expression [23]. Another interesting study should be mentioned here in terms of the groups studied. The study by Kovač et al. included the “all” group of ovarian serous cystadenocarcinoma patients and the “MSC: enriched” group (with samples with a high content of mesenchymal stem cells) [30]. They found that higher miR-107 expression was significantly associated with better OS in both the “all” and “MSC: enriched” groups. It was also observed that higher expression of miR-103a-3p was significantly associated with better OS but in the “MSC: enriched” group [30]. A group of ovarian serous cystadenocarcinoma patients also comprised a cohort in the study by Zhang et al., where higher miR-363-3p expression was found to be significantly associated with better OS and PFS [31]. Not only single miRNA molecules, but also pairs or groups of molecules show their potential as prognostic markers. Thus, a study by Gahlawat et al. evaluated the total circulating cell-free microRNA (cf-miRNA) [32]. This study found that higher levels of cf-miRNA were significantly associated with OS, but not with PFS. Patients with high cf-miRNA levels showed a shorter OS [32]. In the search for prognostic markers in ovarian cancer, miRNA–mRNA pairs were also studied. To determine the contribution of miRNA–mRNA pairs to OS, the authors classified patients into two groups: those at a “high risk” and “low risk” of a short OS [33]. The results of the study showed that “high-risk” patients had a lower OS compared to “low-risk” patients for the following miRNA–mRNA pairs: hsa-miR-126-3p~PROCR (*p* < 0.0001), hsa-miR-223-3p~HBEGF (*p* < 0.0001), hsa-miR-223-3p~CH25H (*p* < 0.0001), hsa-miR-223-3p~NAMPT (*p* < 0.0001), hsa-miR-23a-5 p~ATF3 (*p* < 0.0001), hsa-miR-23a-5 p~HBEGF (*p* < 0.0001), hsa-miR-27a-5 p~EMP1 (*p* = 0.0019), hsa-miR-27a-5 p~ATF3 (*p* < 0.0001), hsa-miR-27a-5 p~HBEGF (*p* = 0.00051), hsa-miR-486-5 p~ATF3 (*p* < 0.0001), hsa-miR-486-5 p~HBB (*p* = 0.00014), and hsa-miR-5 06-3p~POSTN (*p* = 0.00035) [33]. The microRNA studied in combination with another marker in assessing the prognosis of ovarian cancer was miR-338-3p [34,35]. The study by Zhang et al. examined miR-338-3p and *MACC1* gene expression in patients with EOC [34]. The expression of miR-338-3p and MACC1 was divided according to the median into relatively high and relatively low. Thus, four subgroups were created: miR-338-3p High MACC1 High, miR-338-3p Low MACC1 High, miR-338-3p High MACC1 Low, and miR-338-3p Low MACC1 Low. Overall survival and progression-free survival were evaluated. The overall survival of patients with low miR-338-3p expression and high MACC1 expression was shorter than that of patients with high miR-338-3p expression and high MACC1 expression, with high miR-338-3p expression and low MACC1 expression, and with low miR-338-3p expression and low MACC1 expression (*p* = 7.219 × 10^−5^). Furthermore, PFS was also shortest in the group of patients with low expression of miR-338-3p and high expression of MACC1 (*p* = 2.828 × 10^−5^) [34]. Similarly, the expression of miR-338-3p and PURPL were evaluated as prognostic markers in ovarian cancer [35]. This study also distinguished four groups considering the expression of the biomarkers studied: PURPL Low miR-338-3p High, PURPL High miR-338-3p Low, PURPL High miR-338-3p High, and PURPL Low miR-338-3p Low. Overall survival (OS) and recurrence-free survival (RFS) were assessed. Patients with high PURPL expression and low miR-338-3p expression had both a worse OS (*p* = 0.0005) and RFS (*p* = 0.0002) compared to the other groups [35].

**Figure 1:**
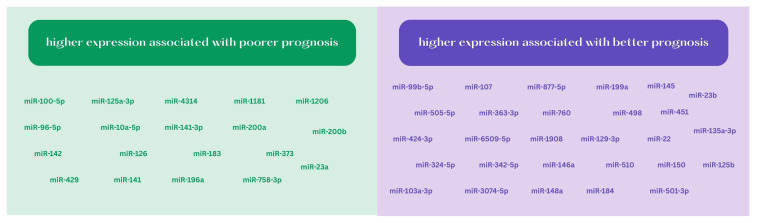
The relevance of single miRNAs in the prognosis of ovarian cancer.

Thus, as a review of recent years shows, microRNAs are important prognostic biomarkers in patients with ovarian cancer. Both the evaluation of single miRNA molecules and miRNAs in combinations with other markers, or circulating cell-free microRNAs, seem useful. Newer and newer studies are discovering and defining these molecules as important factors associated with survival. The relevance of single miRNAs in the prognosis of ovarian cancer is shown in Table 1 and Figure 1.

**Figure 2:**
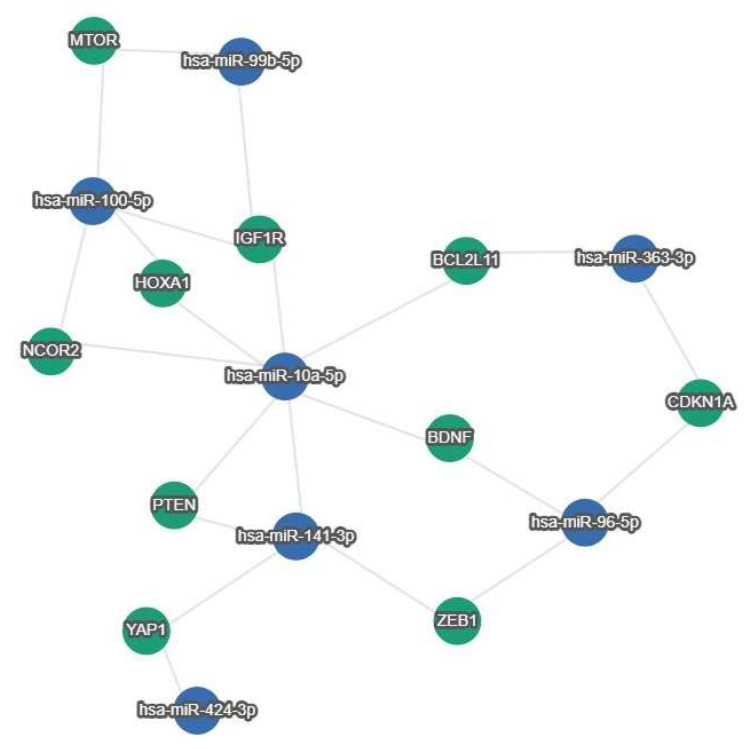
MiRTargetLink 2.0 Gene Interaction Pathway. This pathway visualizes predicted strong interactions between 2 or more miRNAs for each target gene.

Figure 2 shows the interactions between miRNAs described in ovarian cancer prognosis and genes (Figure 2).

## 3. The Role of miRNAs in the Diagnosis of OC

Also of clinical importance is the use of miRNAs in the diagnosis of ovarian cancer. Currently, there are no specific markers for ovarian cancer, which is why it is so difficult to detect this cancer. Thus, it seems important to look for new markers that would distinguish ovarian cancer from benign ovarian tumors or from healthy controls. It would seem particularly important to find a marker that detects ovarian cancer at an early stage. The invention of a screening marker would accelerate the referral of a patient to the diagnostic and therapeutic pathway, which could consequently affect treatment and prognosis. In the face of research in recent years, microRNAs represent hope, as is presented in this review. MicroRNAs are such a large group of molecules that, to date, most studies have described the different miRNAs in ovarian cancer diagnosis one at a time. Of course, they bring important clinical data, but it seems necessary to study the same molecules on multiple cohorts to confirm the results. To date, a well-described miRNA appears to be miR-451a. The diagnostic significance of miR-451a has been demonstrated in differentiating patients with malignant pelvic masses from patients with benign ovarian tumors (AUC 0.62), and the sample tested was plasma [55]. Using the ROC curve, the AUC is supposed to be above 0.5 so that the test can distinguish between the two groups. Therefore, the test is expected to have an AUC as close to 1 as possible. In the study by Záveský et al., miR-451a was tested in different samples [56]. They compared ovarian cancer with normal ovaries, where the test material was tissue, and found the validity of miR-451a as a marker (AUC 0.974). The utility of this marker was also found by comparing ascitic fluid from ovarian cancer patients with plasma from healthy controls (AUC 0.987) [56]. Other miRNAs that have been studied in ovarian cancer several times should be described here. The validity of miRNA-145 as a diagnostic marker to differentiate ovarian cancer from healthy controls was established, where serum was the test material [22,57]. MiRNA-145-5p was also evaluated, and by examining plasma, the utility of this marker was also demonstrated in differentiating ovarian cancer from normal controls (AUC 0.702) [58]. Ovarian cancer diagnostic markers tested in the blood are also miR-205-5p [58,59], miR-346 [58,59], and miR-125b [43,60]. Currently, most of the markers used in clinical practice are assessed in the blood, so studies showing the validity of blood biomarkers appear to be particularly important. However, the possibility of determining miRNA biomarkers in urine should also be noted. The urine collection procedure is less invasive than blood collection and would perhaps provide an alternative sample of material for biomarker determination in the future. A diagnostic marker for ovarian cancer assessed in both serum and urine is miR-6076 [61,62]. Another microRNA tested in urine that distinguished ovarian serous adenocarcinoma from healthy controls is miR-30a-5p [62]. As in prognostics, the miRNA-200 family also plays an important role in diagnosis. MiR-200a as a diagnostic marker for ovarian cancer was tested in both tissue (AUC 0.8088) and serum (AUC 0.8063) [18]. The same was true for miR-200b, where the AUC was 0.8425 for tissue and 0.8625 for serum [18]. This study distinguished between epithelial ovarian cancer and benign ovarian disease or healthy physical examination. The next microRNA of this family to be compared was miR-200b-3p, which was evaluated in tissue. Ovarian cancer and normal ovaries were compared, yielding an AUC of 1.000, and a 95% CI of 0.877–1.000 [56]. MiR-200c was also tested as a serum diagnostic marker for ovarian cancer [21], similarly to miR-200c-3p [59]. In another study, miR-200c-3p was also evaluated in plasma, where patients with malignant pelvic masses and patients with a benign ovarian tumor were compared, and an AUC of 0.78 was obtained [55]. As is shown here, most studies have examined selected miRNAs as diagnostic factors distinguishing ovarian cancer from benign tumors or healthy controls. However, there are also reports comparing slightly different groups. A study by Kumari et al. compared endometrioid ovarian cancer vs. endometriosis and endometrioid endometrial cancer, yielding an AUC of 0.933 [63]. Not only single miRNAs, but also pairs or panels of miRNAs have been studied in the diagnosis of ovarian cancer. The diagnostic potential of combined miR-205 and miR-34a was evaluated, receiving an AUC of 82.7% [64]. In contrast, Gahlawat et al. studied cf-miRNAs [32]. For the diagnostic evaluation of ovarian cancer, they extracted a signature panel of seven cf-miRNAs: miR-92a, miR-200c, miR-320b, miR-320c, miR-335, miR-375, and miR-486. This panel could detect early cancer cases with an AUC of 0.81, but also late cancer cases by increasing the AUC to 0.9 [32]. These are not the only studies evaluating panels of miRNAs in OC diagnosis. Patients with ovarian cancer were distinguished from normal controls by a signature of combination miRNAs: miR-205-5p, miR-145-5p, miR-10a-5p, miR-346, and miR-328-3p (AUC 0.760) [58], but OC was also distinguished from normal controls by a different signature of combined miRNAs: miR-200c-3p, miR-346, miR-127-3p, miR-143-3p, and miR-205-5p (AUC 0.737) [59]. A signature of ten miRNAs was also identified (hsa-miR-1271-5p, hsa-miR-574-3p, hsa-miR182-5p, hsa-miR-183-5p, hsa-miR-96-5p, hsa-miR-15b-5p, hsa-miR-182-3p, hsa-miR-141-5p, hsa-miR-130b-5p, and hsa-miR135b-3p), which was able to differentiate human ovarian cancer tissues from normal tissues [65]. A neural network model was used to evaluate miR-200 members to differentiate ovarian cancer patients and controls. For American patients, the best neural network model consisting of miR-200a/miR-200b/miR-429/miR141 showed an AUC of 0.904, while for Chinese patients, the model consisting of miR-200b/miR-200c/miR-429/miR-141 showed an AUC of 0.901 [66]. The relevance of single miRNAs in the diagnosis of ovarian cancer is shown in Table 2.

**Figure 3:**
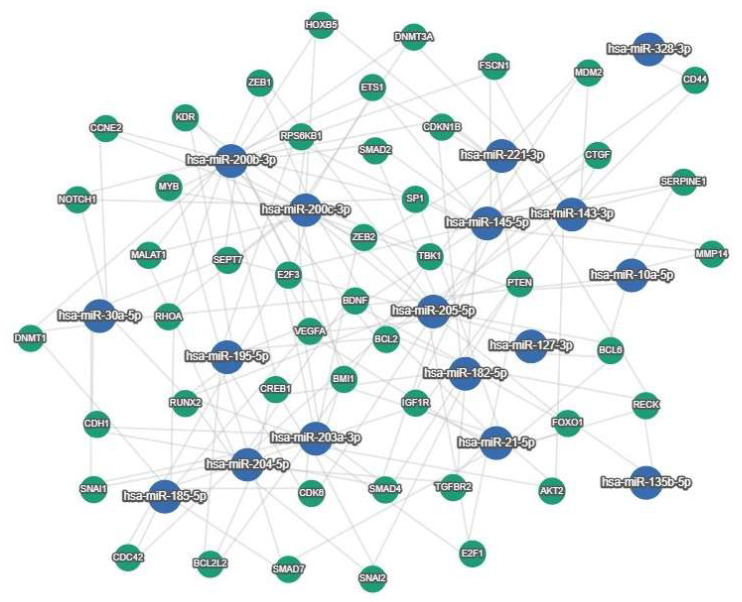
MiRTargetLink 2.0 Gene Interaction Pathway. This pathway visualizes predicted strong interactions between 3 or more miRNAs for each target gene.

Figure 3 shows the interactions between miRNAs described in ovarian cancer diagnosis and genes (Figure 3).

## 4. Summary and Conclusions

**Figure 4:**
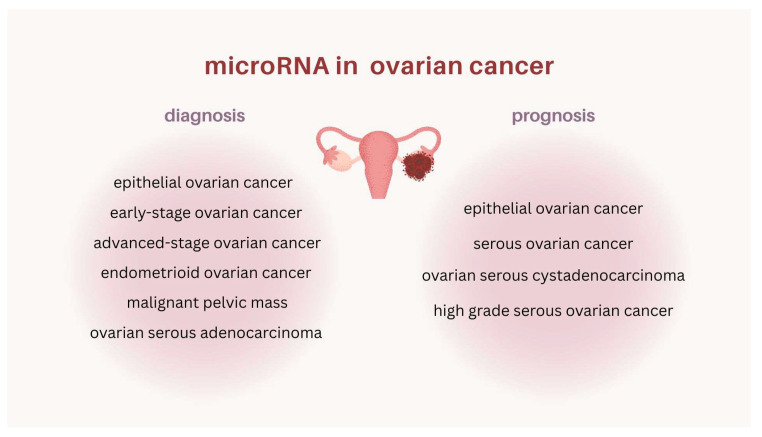
MicroRNAs as biomarkers in the diagnosis and prognosis of ovarian cancer.

MicroRNAs are promising clinical markers in ovarian cancer patients. As is shown here, they are important in the prognosis as well as diagnosis of OC. MiRNAs are a large group of molecules, and research on them is still ongoing. It should be noted that most of the miRNAs reviewed here have been studied once, so despite the promising results obtained for the population, it seems necessary to conduct studies to confirm or exclude the importance of specific miRNAs in ovarian cancer. As is demonstrated, the expression (high or low) of various miRNAs was differentially associated with survival in OC patients, with some miRNAs being associated with a longer survival and some with a shorter survival. Indirectly, this may indicate different functions of miRNAs, which translates into clinical outcomes. These differences may also be due to the different methodologies of the various studies. In the absence of diagnostic markers for OC, the raised role of miRNAs in diagnosis seems all the more important. The diagnostic value of miRNAs has been shown, mostly as blood biomarkers, although they have also been evaluated as tissue or urine markers. MiRNAs have an important role as clinical biomarkers for ovarian cancer, not only as single molecules, but also as biomarker pairs (e.g., miRNA–mRNA) or panels of miRNAs. They are markers both for epithelial ovarian cancer in general, but also for selected subtypes (histological and clinical) of ovarian cancer (Figure 4).

## Figures and Tables

**Table 1:** Association of miRNA expression with survival in ovarian cancer patients.

miRNAs	Survival	Cohort	Compared Expression Groups	Reference
miR-99b-5p	OS: better OS, *p* = 0.011 PFI: better PFI, *p* = 0.0025	Serous ovarian cancer	High vs. low	[28]
miR-100-5p	PFI: shorter PFI, *p* = 0.0088	Serous ovarian cancer	High vs. low	[28]
miR-125a-3p	OS: shorter OS, *p* = 0.039	Serous ovarian cancer	High vs. low	[28]
miR-505-5p	OS: better OS, *p* = 0.0009	Serous ovarian cancer	High vs. low	[28]
miR-424-3p	OS: better OS, *p* = 0.021 PFI: better PFI, *p* = 0.044	Serous ovarian cancer	High vs. low	[28]
miR-324-5p	OS: better OS, *p* = 0.0048	Serous ovarian cancer	High vs. low	[28]
miR-4314	OS: shorter OS, *p* = 0.007 DFS: shorter DFS, *p* < 0.004	Epithelial ovarian cancer	High vs. low	[36]
miR-1181	OS: shorter OS, *p* < 0.001 DFS: shorter DFS, *p* < 0.001	Epithelial ovarian cancer	High vs. low	[36]
miR-1206	OS: shorter OS, *p* = 7.1 × 10^−7^	Ovarian cancer	High vs. low	[37]
miR-96-5p	OS: shorter OS, *p* = 0.0026	Ovarian cancer	High vs. low	[37]
miR-10a-5p	OS: shorter OS, *p* = 0.021	Ovarian cancer	High vs. low	[37]
miR-141-3p	OS: shorter OS, *p* = 0.046	Ovarian cancer	High vs. low	[37]
miR-103a–3p	OS: better OS, *p* = 0.015	Ovarian serous cystadenocarcinoma—MSC: enriched	High vs. low	[30]
miR-107	OS: better OS, *p* = 0.0039	Ovarian serous cystadenocarcinoma—all patients	High vs. low	[30]
miR-107	OS: better OS, *p* = 0.0067	Ovarian serous cystadenocarcinoma—MSC: enriched	High vs. low	[30]
miR-363-3p	OS: better OS, *p* = 0.0060 PFS: better PFS, *p* = 0.0284	Ovarian serous cystadenocarcinoma	High vs. low	[31]
miR-126	OS: shorter OS, *p* = 0.006 RFS: shorter RFS, *p* = 0.007	Epithelial ovarian cancer	High vs. low	[38]
miR-6509-5p	OS: better OS, *p* = 0.006	Ovarian cancer	High vs. low	[39]
miR-342-5p	OS: better OS, *p* = 0.032	Ovarian cancer	High vs. low	[39]
miR-3074-5p	OS: better OS, *p* = 0.015	Ovarian cancer	High vs. low	[39]
miR-877-5p	OS: better OS, *p* = 0.021	Ovarian cancer	High vs. low	[39]
miR-760	OS: better OS, *p* = 0.020	Ovarian cancer	High vs. low	[39]
miR-758-3p	OS: shorter OS, *p* < 0.001	Ovarian cancer	High vs. low	[39]
miR-200a	OS: shorter OS, *p* = 0.0047 DFS: shorter DFS, *p* = 0.0187	Epithelial ovarian cancer	High vs. low	[18]
miR-200b	OS: shorter OS, *p* = 0.0232 DFS: shorter DFS, *p* = 0.0364	Epithelial ovarian cancer	High vs. low	[18]
miR-25	OS: better OS, *p* = 0.004 PFS: better PFS, *p* = 0.005	Ovarian cancer	High vs. low	[26]
miR-142	OS: shorter OS, *p* = 0.049	Ovarian cancer	High vs. low	[26]
miR-501-3p	OS: better OS, *p* = 0.02 DSS: better DSS, *p* = 0.038	Ovarian cancer	High vs. low	[40]
miR-200b	OS: shorter OS, *p* = 0.019	Ovarian cancer	High vs. low	[19]
miR-23a	OS: shorter OS, *p* < 0.01	Ovarian epithelial cancer	High vs. low	[25]
miR-23b	OS: better OS, *p* < 0.01	Ovarian epithelial cancer	High vs. low	[25]
miR-1908	OS: better OS, *p* = 0.004 DFS: better DFS, *p* < 0.001	Ovarian cancer	High vs. low	[41]
miR-146a	Survival: better survival, *p* = 0.003	Advanced serous ovarian cancer	High vs. low	[29]
miR-135a-3p	PFS: better PFS, *p* = 0.0494	Ovarian cancer	High vs. low	[42]
miR-125b	PFS: better PFS, *p* = 0.035	Epithelial ovarian cancer	High vs. low	[43]
miR-148a	OS: better OS, *p* = 0.002	Ovarian cancer	High vs. low	[44]
miR-199a	OS: better OS, *p* = 0.03	Epithelial ovarian cancer	High vs. low	[45]
miR-183	OS: shorter OS, *p* < 0.05	Epithelial ovarian cancer	High vs. low	[46]
miR-373	OS: shorter OS, *p* = 0.033	Epithelial ovarian cancer	High vs. low	[20]
miR-200b	OS: shorter OS, *p* = 0.007	Epithelial ovarian cancer	High vs. low	[20]
miR-200c	OS: shorter OS, *p* = 0.017 DFS: shorter DFS, *p* = 0.019	Epithelial ovarian cancer	High vs. low	[20]
miR-498	OS: better OS, *p* = 0.0056 PFS: better PFS, *p* = 0.003	Ovarian cancer	High vs. low	[47]
miR-129-3p	OS: better OS, *p* = 0.039	Epithelial ovarian cancer	High vs. low	[48]
miR-510	OS: better OS, *p* = 0.048	Epithelial ovarian cancer	High vs. low	[48]
miR-429	OS: shorter, *p* = 0.011	Epithelial ovarian cancer	High vs. low	[49]
miR-184	OS: better OS, *p* < 0.001	Epithelial ovarian cancer	High vs. low	[50]
miR-145	OS: better OS, *p* = 0.023	Malignant ovarian cancer	High vs. low	[22]
miR-200c	OS: better OS, *p* < 0.001	Ovarian cancer	High vs. low	[21]
miR-141	OS: shorter OS, *p* = 0.049	Ovarian cancer	High vs. low	[21]
miR-196a	OS: shorter OS, *p* < 0.001 recurrent-free survival: shorter recurrent-free survival, *p* = 0.003	Ovarian carcinoma	High vs. low	[51]
miR-145	OS: better OS, *p* = 0.003	High-grade ovarian serous carcinoma	High vs. low	[23]
miR-451	OS: better OS, *p* < 0.001	Epithelial ovarian cancer	High vs. low	[52]
miR-25	OS: shorter OS, *p* = 0.001	Epithelial ovarian cancer	High vs. low	[27]
miR-22	OS: better OS, *p* = 0.005 PFS: better PFS, *p* = 0.004	Epithelial ovarian cancer	High vs. low	[53]
miR-150	OS: better OS, *p* < 0.001 PFS: better PFS, *p* < 0.001	Epithelial ovarian cancer	High vs. low	[54]
miR-23b	OS: better OS, *p* < 0.001 PFS: better PFS, *p* < 0.001	Epithelial ovarian cancer	High vs. low	[24]

OS—overall survival, OC—ovarian cancer, PFI—progression-free interval, DFS—disease-free survival, MSC: enriched—samples with high content of mesenchymal stem cells; RFS—relapse-free survival, PFS—progression-free survival, DSS—disease-specific survival.

**Table 2:** Importance of single miRNAs in the diagnosis of ovarian cancer.

miRNAs	AUC (95% CI)	Compared Cohorts	Test Sample	Reference
miR-3653-3p	0.833 (0.779–0.887)	Ovarian cancer vs. healthy controls	PBMCs	[67]
miR-4314	0.78 (0.69–0.85)	Epithelial ovarian cancer vs. healthy controls	Serum	[36]
miR-1181	0.76 (0.67-0.86)	Epithelial ovarian cancer vs. healthy controls	Serum	[36]
miR-1	0.531	Malignant vs. benign ovarian tumors	Serum	[68]
miR-21	0.648	Malignant vs. benign ovarian tumors	Serum	[68]
miR-204	0.924 (0.866–0.982)	Early ovarian cancer	Serum	[69]
	0.942 (0.893–0.990)	Late ovarian cancer		
miRNA-34a	0.97 (0.932–1.008)	Advanced-stage epithelial ovarian cancer	Tissue	[70]
	0.92 (0.842–0.99)	Advanced-stage epithelial ovarian cancer	Serum	
	0.969 (0.938–1.001)	Early-stage epithelial ovarian cancer	Tissue	
	0.827 (0.628–0.95)	Early-stage epithelial ovarian cancer	Serum	
miRNA-let-7f	0.921 (0.853–0.989)	Advanced-stage epithelial ovarian cancer	Tissue	[70]
	0.879 (0.773–0.98)	Advanced-stage epithelial ovarian cancer	Serum	
	0.871 (0.788–0.954)	Early-stage epithelial ovarian cancer	Tissue	
	0.82 (0.677–0.96)	Early-stage epithelial ovarian cancer	Serum	
miRNA-31	0.921 (0.725–0.949)	Advanced-stage epithelial ovarian cancer	Tissue	[70]
	0.856 (0.694–1.01)	Advanced-stage epithelial ovarian cancer	Serum	
	0.866 (0.766–0.969)	Early-stage epithelial ovarian cancer	Tissue	
	0.81 (0.642–0.97)	Early-stage epithelial ovarian cancer	Serum	
miRNA-200a	0.8088 (0.6749–0.9426)	Epithelial ovarian cancer vs. benign ovarian disease or healthy physical examination	Tissue	[18]
	0.8063 (0.6745–0.9380)	Epithelial ovarian cancer vs. benign ovarian disease or healthy physical examination	Serum	
miRNA-200b	0.8425 (0.7197–0.9653)	Epithelial ovarian cancer vs. benign ovarian disease or healthy physical examination	Tissue	[18]
	0.8625 (0.7459–0.9791)	Epithelial ovarian cancer vs. benign ovarian disease or healthy physical examination	Serum	
miR-1290	0.988	EOC vs. benign ovarian neoplasm	Tissue	[71]
	0.794	EOC vs. benign ovarian neoplasm	Serum	
miR-1260a	0.660 (0.588–0.733)	Ovarian cancer vs. healthy control	Peripheral blood lymphocytes	[72]
miR-1260b	0.704 (0.635–0.773)	Ovarian cancer vs. healthy control	Peripheral blood lymphocytes	[72]
miR-143	0.933 (0.842–1.000)	Endometrioid ovarian cancer vs. endometriosis and endometrioid endometrial cancer	Tissue	[63]
miR-145	0.928 (0.86–0.95)	Epithelial ovarian cancer vs. healthy controls	Serum	[57]
miR-361-3p	0.838	Ovarian cancer vs. control group and patients with benign mass	Serum	[73]
miR-200c-3p	0.78	Malignant pelvic mass vs. patients with a benign ovarian tumor	Plasma	[55]
miR-221-3p	0.65	Malignant pelvic mass vs. patients with a benign ovarian tumor	Plasma	[55]
miR-195-5p	0.63	Malignant pelvic mass vs. patients with a benign ovarian tumor	Plasma	[55]
miR-21-5p	0.63	Malignant pelvic mass vs. patients with a benign ovarian tumor	Plasma	[55]
miR-451a	0.62	Malignant pelvic mass vs. patients with a benign ovarian tumor	Plasma	[55]
miR-484	0.63	Malignant pelvic mass vs. patients with a benign ovarian tumor	Plasma	[55]
miR-205-5p	0.681	Ovarian cancer vs. normal controls	Plasma	[58]
miR-145-5p	0.702	Ovarian cancer vs. normal controls	Plasma	[58]
miR-10a-5p	0.680	Ovarian cancer vs. normal controls	Plasma	[58]
miR-346	0.737	Ovarian cancer vs. normal controls	Plasma	[58]
miR-328-3p	0.700	Ovarian cancer vs. normal controls	Plasma	[58]
miR-200c-3p	0.726	Ovarian cancer vs. normal controls	Serum	[59]
miR-346	0.693	Ovarian cancer vs. normal controls	Serum	[59]
miR-127-3p	0.698	Ovarian cancer vs. normal controls	Serum	[59]
miR-143-3p	0.687	Ovarian cancer vs. normal controls	Serum	[59]
miR-205-5p	0.689	Ovarian cancer vs. normal controls	Serum	[59]
miR-200b-3p	1.000 (0.877–1.000)	Ovarian cancer vs. normal ovary	Tissue	[56]
miR-182-5p	0.995 (0.867–1.000)	Ovarian cancer vs. normal ovary	Tissue	[56]
miR-135b-5p	0.847 (0.661–0.954)	Ovarian cancer vs. normal ovary	Tissue	[56]
miR-451a	0.974 (0.832–1.000)	Ovarian cancer vs. normal ovary	Tissue	[56]
miR-204-5p	0.934 (0.772–0.993)	Ovarian cancer vs. normal ovary	Tissue	[56]
miR-185-5p	0.811 (0.619–0.933)	Ovarian cancer vs. normal ovary	Tissue	[56]
miR-203a-3p	0.765 (0.568–0.904)	Ovarian cancer vs. normal ovary	Tissue	[56]
miR-203a-3p	1.000 (0.858–1.000)	Ovarian cancer vs. healthy controls	Ascitic fluid (ovarian cancer), plasma (healthy controls)	[56]
miR-204-5p	1.000 (0.858–1.000)	Ovarian cancer vs. healthy controls	Ascitic fluid (ovarian cancer), plasma (healthy controls)	[56]
miR-135b-5p	1.000 (0.858–1.000)	Ovarian cancer vs. healthy controls	Ascitic fluid (ovarian cancer), plasma (healthy controls)	[56]
miR-451a	0.986 (0.833–1.000)	Ovarian cancer vs. healthy controls	Ascitic fluid (ovarian cancer), plasma (healthy controls)	[56]
miR-182-5p	0.986 (0.833–1.000)	Ovarian cancer vs. healthy controls	Ascitic fluid (ovarian cancer), plasma (healthy controls)	[56]
miR-1273g-3p	0.7	Recurrent epithelial ovarian cancer vs. healthy controls	Serum	[74]
miR-320a	0.96 (0.95–0.98)	Ovarian cancer vs. non-cancer	Serum	[61]
miR-665	0.86 (0.82–0.89)	Ovarian cancer vs. non-cancer	Serum	[61]
miR-3184-5p	0.97 (0.96–0.98)	Ovarian cancer vs. non-cancer	Serum	[61]
miR-6717-5p	0.73 (0.68–0.78)	Ovarian cancer vs. non-cancer	Serum	[61]
miR-4459	0.61 (0.56–0.65)	Ovarian cancer vs. non-cancer	Serum	[61]
miR-6076	0.56 (0.51–0.61)	Ovarian cancer vs. non-cancer	Serum	[61]
miR-3195	0.83 (0.79–0.87)	Ovarian cancer vs. non-cancer	Serum	[61]
miR-1275	0.87 (0.84–0.91)	Ovarian cancer vs. non-cancer	Serum	[61]
miR-3185	0.70 (0.65–0.75)	Ovarian cancer vs. non-cancer	Serum	[61]
miR-4640-5p	0.54 (0.48–0.61)	Ovarian cancer vs. non-cancer	Serum	[61]
miR-125b	0.737	Epithelial ovarian cancer vs. benign ovarian tumor	Serum	[43]
miR-199a	0.704	Epithelial ovarian cancer vs. healthy controls	Serum	[45]
miR-125b	0.728 (0.64–0.81)	Epithelial ovarian cancer vs. healthy controls	Serum	[60]
miR-145	0.82 (0.77–0.88)	Malignant ovarian cancer vs. healthy controls	Serum	[22]
miR-30a-5p	0.862 (0.709–1.016)	Ovarian serous adenocarcinoma vs. healthy controls	Urine	[62]
miR-6076	0.693 (0.482–0.904)	Ovarian serous adenocarcinoma vs. healthy controls	Urine	[62]
miR-200c	0.79 (0.71–0.87)	Ovarian cancer vs. healthy controls	Serum	[21]
miR-141	0.75 (0.67–0.83)	Ovarian cancer vs. healthy controls	Serum	[21]

PBMCs—peripheral blood mononuclear cells.
